# Morphology and size control of an amorphous conjugated polymer network containing quinone and pyrrole moieties *via* precipitation polymerization†

**DOI:** 10.1039/d3na01006f

**Published:** 2023-12-27

**Authors:** Ryuto Sugiura, Hiroaki Imai, Yuya Oaki

**Affiliations:** a Department of Applied Chemistry, Faculty of Science and Technology, Keio University 3-14-1 Hiyoshi, Kohoku-ku Yokohama 223-8522 Japan oakiyuya@applc.keio.ac.jp

## Abstract

Morphology and size control of insoluble and infusible conjugated polymers are significant for their applications. Development of a precipitation polymerization route without using a surface stabilizer is preferred to control the reaction, morphology, and size. In the present work, precipitation polymerization for an amorphous conjugated polymer network, a new type of polymerized structure containing functional units, was studied for the size and morphology control in the solution phase at low temperature. The random copolymerization of benzoquinone (BQ) and pyrrole (Py) monomers formed microspheres of the BQ–Py network polymers as the precipitates in the solution phase. The particle diameter was controlled in the range of 70 nm and 1 μm by changing the pH of the solution and concentration of the monomers. The resultant nanoparticles were applied to a metal-free electrocatalyst for the hydrogen evolution reaction (HER). The catalytic activity of the BQ–Py nanoparticles was higher than that of the bulk micrometer-sized particles. The results imply that the morphology and size of amorphous conjugated polymer networks can be controlled by precipitation polymerization.

## Abstract 2

The precipitation polymerization method, an efficient stabilizer-free route to obtain polymer microspheres, was developed for the size and morphology control of an amorphous conjugated polymer network containing benzoquinone and pyrrole moieties.

## Graphical abstract

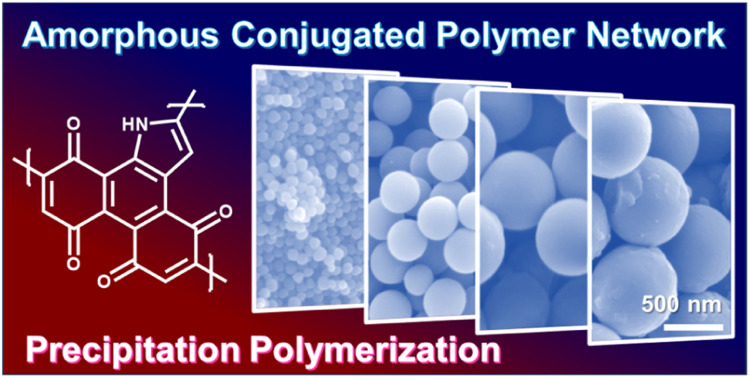

## Introduction

**Fig. 1:**
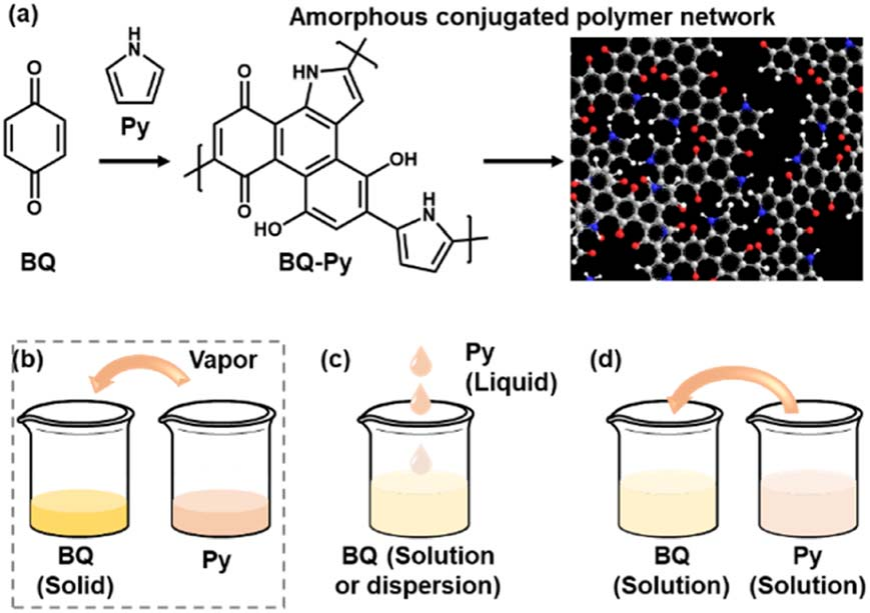
Schematic illustration of a BQ–Py amorphous conjugated polymer network (a) and its synthetic methods (b–d). (a) Proposed synthetic route and structure of the BQ–Py polymer. (b) Solid–vapor synthesis in our previous work.^13^ (c) Liquid-phase synthesis. (d) Solution synthesis for precipitation polymerization.

**Scheme 1:**
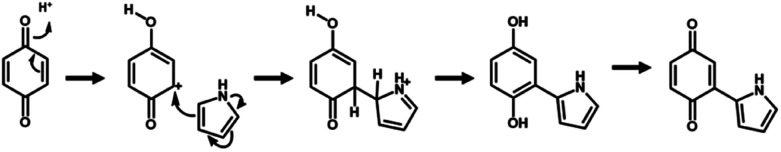
Electrophilic substitution of BQ and Py for polymerization.

**Scheme 2:**
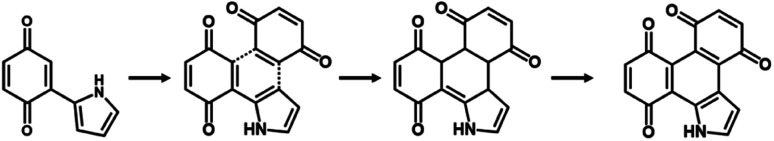
Pericyclic reaction of the BQ–Py diad and BQ.

Polymerized structures of functional molecules have a variety of types, such as main- and side-chain, ladder, and two-dimensional structures.^1–7^ In recent years, crystalline porous frameworks, such as metal organic and covalent organic frameworks (MOFs and COFs), have attracted much interest as new nanoarchitectures.^8–11^ Assembly states of the unit molecules have effects on the structural flexibility, *i.e.* dynamic molecular and segmental motions, and properties. When π-conjugated functional units are introduced in the macromolecular assemblies, the π–π stacking easily provides a rigid and/or crystalline structure with a lower flexibility. Our group has proposed an amorphous conjugated polymer network as a new type of polymerized structure.^12–14^ Functional conjugated moieties are introduced in a structurally flexible and random polymer network with their isolated and dispersed states. When multiple polymerization reactions simultaneously proceed with a number of monomers, randomly copolymerized networks are obtained spontaneously (Fig. 1a). For example, benzoquinone (BQ) and pyrrole (Py) formed a network polymer *via* the C–C bond formation and pericyclic reaction (Schemes 1 and 2). The BQ–Py polymers were used for electrochemical applications, such as in a HER catalyst, battery, and photoanode, by our group and other groups.^13,15–18^ However, the size and morphology were not controlled in the previous studies because the polymerization was performed in an inhomogeneous system, such as a combination of a BQ solid and Py vapor (Fig. 1b). In recent years, the size and morphology control of conjugated polymers in microscopic and macroscopic scales has attracted much interest for their applications.^19–23^ In the present work, the precipitation polymerization of BQ–Py in the solution phase was studied to control the reaction, size, and morphology (Fig. 1c and d). The controllable polymerization method can be applied to syntheses and applications of various amorphous conjugated polymer networks.

The BQ–Py polymer network formed the stacking of the quasi-graphitic structures.^13^ The bulk particles with a layered structure were exfoliated into the nanosheets. Layered structures and their exfoliated nanosheets have attracted much interest as two-dimensional (2D) materials.^24–30^ These 2D materials generally exhibit the platy and flake-like shapes originating from the intrinsic anisotropy of the unit layers. The morphology control of the anisotropic layered materials to isotropic shapes, such as spheres and cubes, was not easily achieved by the conventional synthetic routes. In previous studies, isotropic morphologies were obtained by the conversion of spherical amorphous precursors.^31,32^ As another method, the rapid reaction and precipitation of the layered metal oxides provided the nanoparticles with the inhibition of the growth.^33–36^ The results imply that the nucleation burst at the initial stage of the reaction in the solution phase can form spherical nanoparticles even with the intrinsically anisotropic structures. In the present work, the size control of BQ–Py spherical particles with a graphitic layered structure was achieved by precipitation polymerization of the BQ and Py monomers in the solution phase. The polymerization rate was controlled by using the concentration of proton and monomers. The spherical nanoparticles in the range of 70 nm and 1 μm were obtained by changes in the monomer concentration and pH. The nanoparticles *ca.* 100 nm in size showed improved HER catalytic activity compared with that of the irregular shaped bulky particles.

## Results and discussion

### Solvents for the solution-phase synthesis

The simultaneous random reactions of BQ and Py provide the network copolymer.^13^ The first step for polymerization is C–C bond formation between the *α* position of BQ and 2 position of Py *via* electrophilic substitution (Scheme 1). Similar reactions between the quinone and pyrrole moieties were reported in earlier studies.^37–39^ The subsequent pericyclic reaction proceeds between the BQ–Py diad and free BQ with dehydrogenation. A similar pericyclic reaction was studied between the indole-naphthoquinone (NQ) diad and free NQ.^40^ A BQ–Py random copolymer network is spontaneously formed by these two reactions with different rates and directions. In our previous work,^13^ Py vapor was supplied to a BQ solid in a sealed chamber at 60 °C under ambient pressure (Fig. 1b). In the present study, the inhomogeneous system was changed to a homogenous system in the liquid phase (Fig. 1c and d). If rapid nucleation of the seeds is achieved in the solution phase, the particle size can be controlled by using the conditions for precipitation polymerization. Rapid reactions for polymerization are required for preparation of the seeds. Therefore, the effects of the solvents on the reaction rate were first studied for the control of the size and morphology. Here eight solvents, namely water, ethanol, 2-propanol, dimethylformamide (DMF), tetrahydrofuran (THF), toluene, acetone, and ethyl acetate, were used to prepare the solution phase. BQ monomer (2.0 mmol) was dissolved (or dispersed for water) in 4 mL of these solvents at 60 °C. The neat Py liquid (2.0 mmol) heated at 60 °C was dropped in the stirred solution (or dispersion liquid) containing BQ. The mixture was maintained at 60 °C for 1 h and then vacuum-dried at 200 °C for 16 h to evaporate the remaining solvents and remove the monomers and oligomers. The detailed methods are described in the ESI.†

**Fig. 2:**
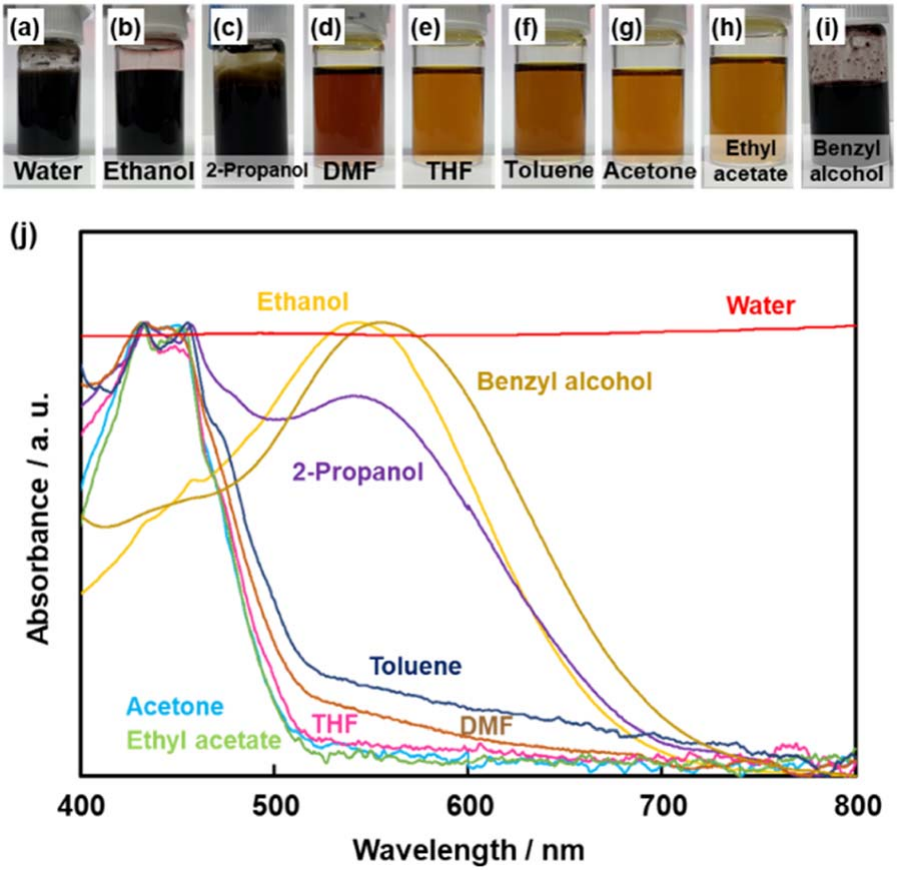
Effect of the solvents on the polymerization behavior of BQ and Py. (a–i) Photographs after polymerization for 1 h in each solvent. (j) UV-vis spectra of the liquid phase after polymerization for 1 h.

A black precipitate was immediately observed upon dropping Py in the BQ aqueous dispersion liquid (Fig. 2a). The yield was 18.1% in the aqueous phase. Although the ethanol and 2-propanol solutions showed a dark-brown color upon mixing (Fig. 2b and c), no precipitate was collected after vacuum drying. The other solutions only showed color changes to brown without precipitation even after 1 h (Fig. 2d–h). UV-vis spectra indicate the polymerization behavior (Fig. 2j). If the polymerization proceeds, absorption is observed in the UV-vis region with the formation of the conjugated structures (Schemes 1 and 2). A broadened absorption peak in the range of 400–500 nm was observed for DMF, THF, toluene, acetone, and ethyl acetate. The absorption peak was shifted to the longer wavelength region for 2-propanol and ethanol. Moreover, the aqueous medium showed the absorption in the entire range of the visible-light region. These results imply that the protonic solvents promote the polymerization of BQ and Py, particularly in the reaction in Scheme 1. When benzyl alcohol as a protonic solvent was used to verify the hypothesis, the color of the solution was changed to dark brown (Fig. 2i). The colloidal liquid showed the broadened absorption centered at 550 nm (Fig. 2j). As the reaction of BQ and Py starts with the protonation of BQ (Scheme 1), the protonic solvents promote the polymerization reactions leading to precipitation. These experiments indicate that the solution synthesis of the BQ–Py polymer requires a protonic solvent.

**Fig. 3:**
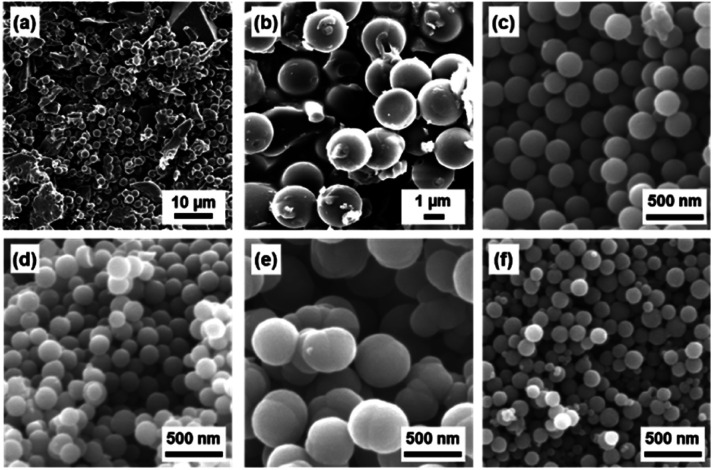
SEM images of the resultant BQ–Py particles in the aqueous (a and b) and acidic solutions (pH 2) adjusted using HNO_3_ (c), CH_3_COOH (d), H_2_SO_4_ (e), and HCl (f).

BQ–Py spherical particles of around 2 μm were observed in the aqueous phase without adjusting the pH (Fig. 3a and b). However, irregularly shaped non-spherical particles were included in the precipitates (Fig. 3a). The proton source and pH in the aqueous phase were changed to increase the polymerization rate for the morphology and size control (Fig. 3c–f). The pH of the aqueous phase was adjusted to 2.0 using acids, namely nitric acid (HNO_3_), acetic acid (CH_3_COOH), sulfuric acid (H_2_SO_4_), and hydrochloric acid (HCl), and then BQ was dispersed in the acid solutions. The average particle size reduced to less than 1 μm in diameter with the addition of the acids (Fig. 3c–f). The yield was improved to 28.9% for HNO_3_, 25.2% for CH_3_COOH, 25.3% for H_2_SO_4_, and 28.3% for HCl. The results support that proton accelerates the polymerization reactions. The smallest particle size in high yield was achieved in the HCl solution (Fig. 3f). However, dropping of neat Py liquid and suspension of BQ cause the local and temporal inhomogeneity of the concentration. Both Py and BQ were dissolved in the solution phase to achieve a more homogeneous system (Fig. 1d), *i.e.* precipitation polymerization.

### Effect of the monomer concentration and pH on the particle size in homogenous solution synthesis

**Fig. 4:**
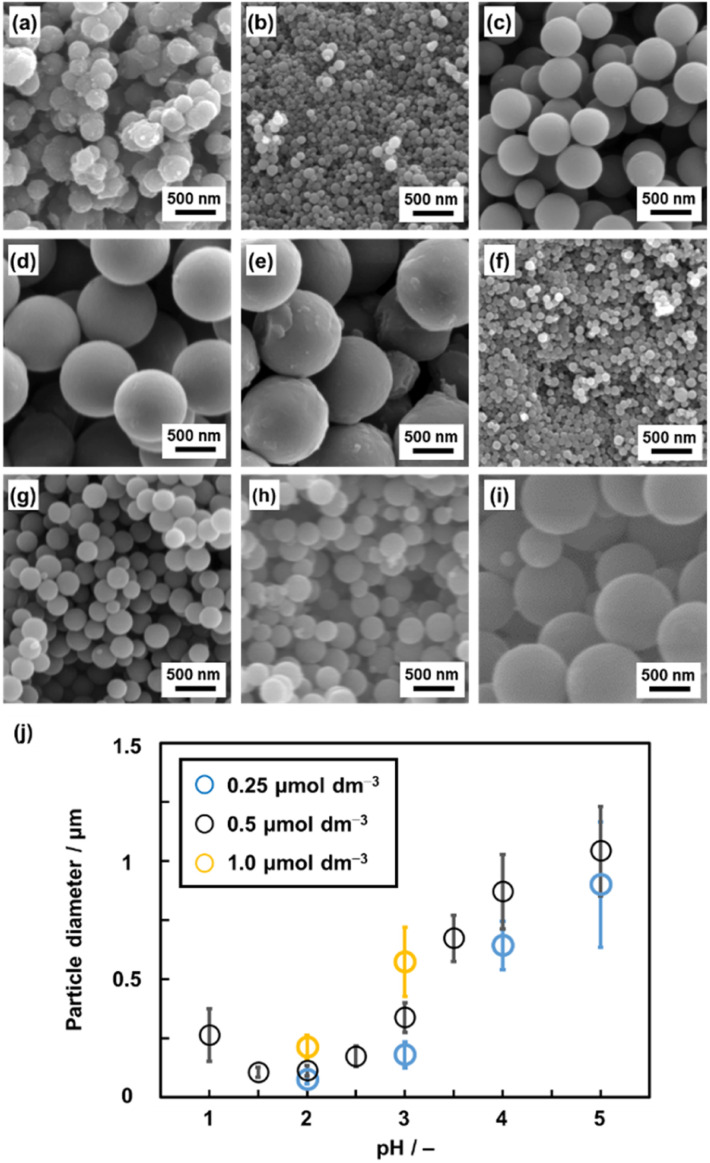
SEM images (a–i) and average diameter (j) of the BQ–Py particles in HCl solution with different pH values and monomer (BQ and Py) concentrations. (a) pH 1.0, 0.5 μmol dm^−3^. (b) pH 2.0, 0.5 μmol dm^−3^. (c) pH 3.0, 0.5 μmol dm^−3^. (d) pH 4.0, 0.5 μmol dm^−3^. (e) pH 5.0, 0.5 μmol dm^−3^. (f) pH 2.0, 0.25 μmol dm^−3^. (g) pH 2.0, 1.0 μmol dm^−3^. (h) pH 3.0, 0.25 μmol dm^−3^. (i) pH 3.0, 1.0 μmol dm^−3^. (j) Relationship between the particle diameter and synthetic conditions (pH and monomer concentrations). The detailed and additional data are in Table S1 and Fig. S1 in the ESI,† respectively.

A homogenous solution system with controlled pH was prepared to achieve particle size control through the growth of the seed particles. Py (2.0 mmol) was dissolved in a solution mixture of ethanol (1 mL) and pH-adjusted acid solution by using HCl (1 mL) at 60 °C. BQ (2.0 mmol) was dissolved in a mixture of ethanol (1 mL) and pH-adjusted acid solution by using HCl (1 mL) at 60 °C. pH of these precursor solutions containing BQ and Py was adjusted to 1.0–5.0 (Fig. 4 and S1 in the ESI†). The precipitate was collected by centrifugation and then vacuum-dried at 200 °C for 16 h. As these monomers were completely dissolved in the solvents, this method is regarded as precipitation polymerization. The spherical particles in the range of 0.1 and 1.0 μm were obtained in a yield of 22–31% (Fig. 4a–e and Table S1 in the ESI†). The smallest and largest particle sizes were 105 ± 22 nm at pH 1.5 and 1042 ± 189 nm at pH 5.0, respectively (Fig. 4b and e).

The particle sizes were 112 ± 23 nm for pH 2.0 and 337 ± 61 nm for pH 3.0 with the standard monomer concentrations (BQ and Py) of 0.5 μmol dm^−3^ (Fig. 4b and c). When the monomer concentrations were decreased to 0.25 μmol dm^−3^, the particle sizes decreased to 73.5 ± 17 nm for pH 2.0 and 179 ± 55 nm for pH 3.0 (Fig. 4f and h). In contrast, the particle size increased to 212 ± 49 nm for pH 2.0 and 573 ± 147 nm for pH 3.0 on increasing the monomer concentrations to 1.0 μmol dm^−3^ (Fig. 4g and i). In this manner, the particle size was controlled with changes in the pH and monomer concentrations of precipitation polymerization (Fig. 4j). In general, precipitation polymerization is used as a stabilizer-free method to obtain microspheres of polymers.^41–45^ Although the polymerization method was applied to vinyl monomers, the syntheses of polymers with conjugated moieties were limited in previous studies.^46–50^ The present work shows that precipitate polymerization can be applied to the size and morphology control of an amorphous conjugated polymer network as a new type of polymer structure.

As polymerization reactions are promoted at a lower pH, the rapid nucleation of the seeds proceeds with the consumption of the monomers. The solubility of the resultant polymer decreases on lowering the monomer concentration. The formation of smaller particles is preferred at a lower pH and monomer concentration (Fig. 4j). In contrast, a higher pH causes a decrease in the number of nuclei and increase in the concentration of the remaining monomer. The solubility of the resultant polymer increases at a higher monomer concentration. The larger particle size forms at a higher pH and monomer concentration (Fig. 4j). These mechanisms are consistent with the scheme of precipitation polymerization in a previous report.^51^

### Structure and composition of the BQ–Py polymer particles

**Fig. 5:**
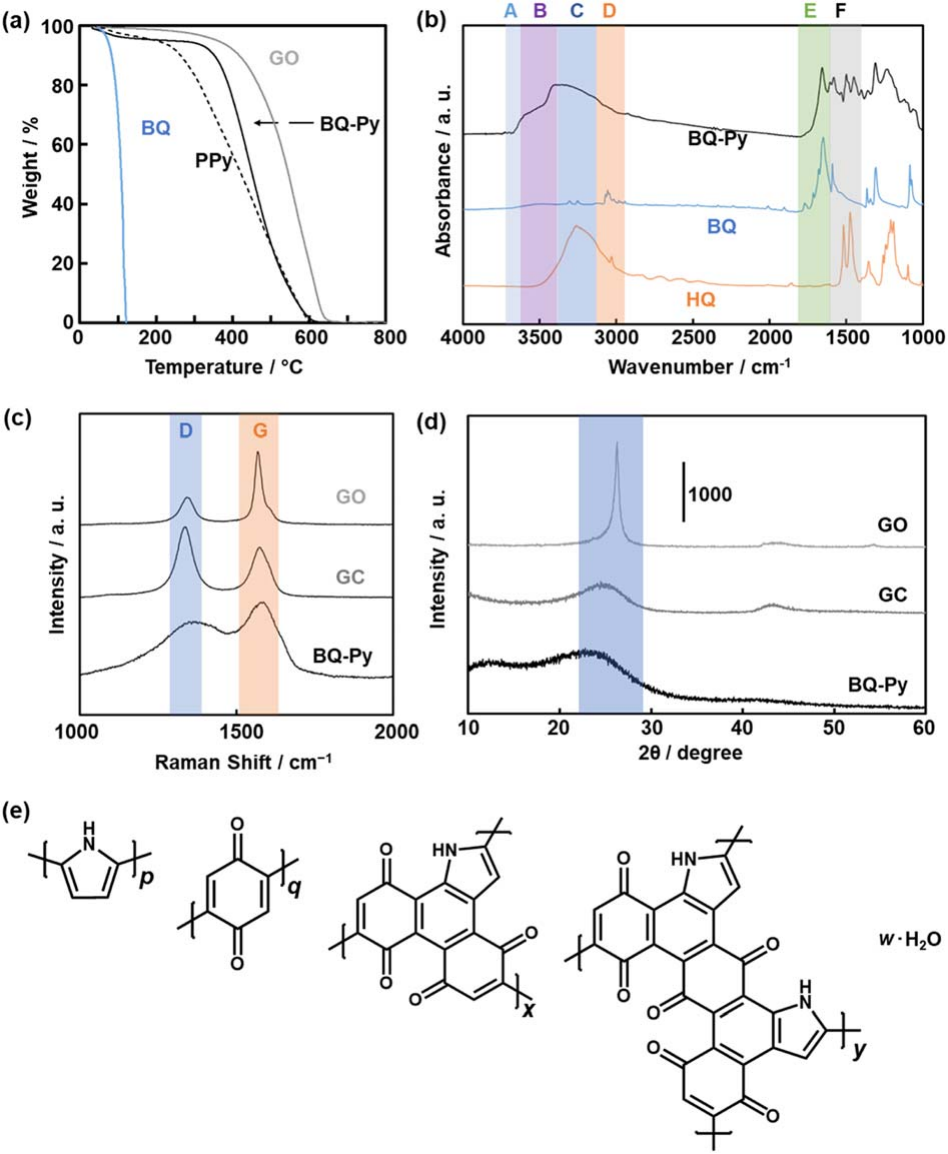
Structural analyses of the BQ–Py nanoparticles synthesized at pH 2.0 and their reference samples. (a) TG curves of the BQ monomer, PPy, BQ–Py, and GO in an air atmosphere. (b) FT-IR spectra of the BQ monomer, HQ, and BQ–Py. (c) Raman spectra of BQ–Py, GC, and GO. (d) XRD patterns of BQ–Py, GC, and GO. (e) Estimated structures of the monomers in the BQ–Py network polymer and hydrated water.

The structure and composition of the BQ–Py polymer particles synthesized at pH 2.0 were similar to those of the bulk BQ–Py in our previous report (Fig. 5).^13^ In thermogravimetry (TG) analysis under an air atmosphere, weight loss was observed in the range of 330–600 °C (Fig. 5a). The temperature range of weight loss was higher than that of a commercial linear polypyrrole (PPy) and lower than that of a commercial graphene oxide (GO) (Fig. 5a). The TG curves have no significant differences in the BQ–Py particles synthesized under different conditions (Fig. S2 in the ESI†). The results indicate that BQ–Py formed a polymer network. The BQ–Py polymer particles show broadened absorption bands of N–H stretching (*ν*_N–H_, band B), C=O stretching (*ν*_C=O_, band E), and C=C stretching (*ν*_C=C_, band F) vibrations in the Fourier-transform infrared spectrum (FT-IR) (Fig. 5b). In addition, a weak absorption tail corresponding to the C–H stretching vibration (*ν*_C–H_, band D) was observed. The absorption band of the O–H stretching vibration (*ν*_O–H_) indicates the hydroquinone (HQ) state with the reduction of the BQ unit (band C) and hydrated water (band A). The appearance of these absorptions is consistent with the estimated structure of the BQ–Py polymer (Fig. 1a). The BQ–Py nanoparticles synthesized under different conditions showed similar FT-IR spectra (Fig. S2 in the ESI†). Formation of the estimated structure with the conjugated polymer network was supported by ^13^C solid-state nuclear magnetic resonance (NMR) (Fig. S3 in the ESI†). These analyses indicate the formation of conjugated polymer networks *via* simultaneous multiple reactions (Fig. 1a, Schemes 1 and 2).

The extension of the π-conjugated framework was studied by Raman and UV-vis-NIR spectroscopies. Raman spectroscopy showed two broadened bands centered at around 1350 and 1590 cm^−1^ corresponding to the D and G bands, respectively (Fig. 5c). The intensity ratio of G to D bands (G/D ratio) was 0.73 for commercial glassy carbon (GC), 1.35 for the BQ–Py nanoparticles *ca.* 100 nm in size synthesized at pH 2.0, and 2.52 for commercial GO. The G/D ratio implies the partial formation of graphitic structures. The broadened spectrum of BQ–Py compared with that of the other samples implies a low crystalline structure. Similar broadened spectra were observed in the other BQ–Py particles with different sizes (Fig. S2 in the ESI†). The UV-vis-NIR spectrum of the BQ–Py particles showed the absorption in the range of 400 and 1200 nm (Fig. S4 in the ESI†), whereas the charge-transfer complex of BQ and HQ had an absorption edge at around 800 nm. These results indicate that the π-conjugated framework is extended with polymerization.

A weakened and broadened halo was observed at around 2*θ* = 25° in the X-ray diffraction (XRD) pattern (Fig. 5d). A lattice spacing (*d*_0_) of 0.39 nm corresponds to the distance between the stacked π-conjugated frameworks. The broadened and weakened diffraction originates from the low-crystalline structure. The other BQ–Py particles with different sizes showed similar broadened and weakened XRD patterns (Fig. S2 in the ESI†). Based on the Raman and XRD analyses, the amorphous BQ–Py network polymer had the low-crystalline stacking of the π-conjugated graphitic structures.

The composition of the BQ–Py polymer network was estimated from CHN elemental analysis (Fig. 5e). The monomer units and compositions (molar ratio) were defined as the linear Py moiety (*p*), BQ moiety (*q*), and two fused-ring structures (*x*, *y*) (*p* + *q* + *x* + *y* = 1). After the water proportion (*w*) was estimated from the weight loss at 200 °C in the TG curve, the other compositions *p*, *q*, *x*, and *y* (molar fraction) were approximated based on the results of the CHN elemental analysis. The ratio of HQ in the reduced state was assumed to adjust the composition. As the weight ratio of C : H : N : O (others) was measured to be 66.5 : 2.81 : 7.68 : 23.0 for the particles synthesized at pH 2.0 (Table S2 in the ESI†), the molar fraction of the repeated monomer units was assumed to be *p* = 0.457, *q* = 0.320, *x* = 0.144, *y* = 0.078, and *w* = 0.381 containing 31.2% of the reduced HQ state. Based on the estimated composition, the calculated weight ratio of C : H : N : O (others) was 66.9 : 3.00 : 7.31 : 22.8. The measured and calculated compositions were consistent with each other within a difference of 0.5%.

**Fig. 6:**
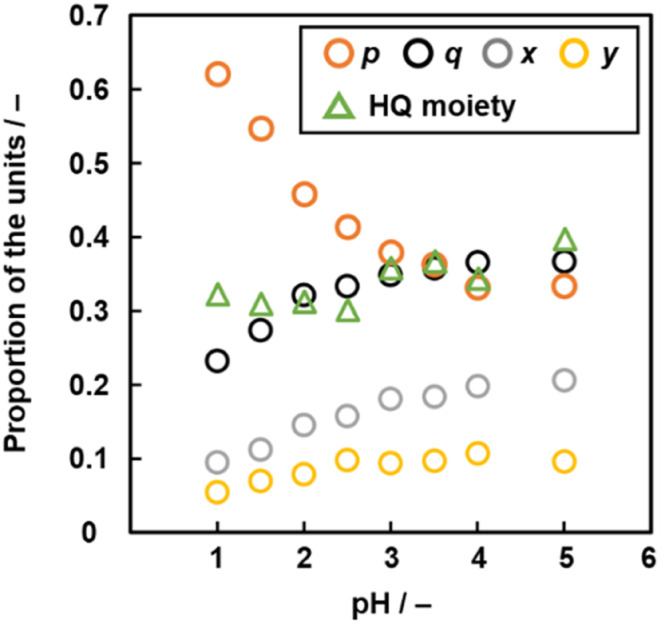
Relationship between pH, molar fraction *p* (Py moiety), *q* (BQ moiety), *x*, *y* (two fused-ring structures, *p* + *q* + *x* + *y* = 1, Fig. 5e) and proportion of the reduced HQ moiety.

The compositions *p*, *q*, *x*, and *y* varied on changes in pH of the solution (Fig. 6 and Table S3 in the ESI†). As the pH of the solution lowered, the ratio of the linearly polymerized Py segments (*p*) and BQ segments (*q*) increased and decreased, respectively. In addition, the ratio of the fused ring structures (*x* and *y*) derived from the pericyclic reaction decreased with lowering pH (Fig. 6). The results are supported by a decrease in the G/D ratio with lowering pH in the Raman spectra (Fig. S2 in the ESI†). As the electrophilic substitution reaction in Scheme 1 is promoted with an increase in protons, the Py proportion increases in the network polymer.

### HER catalytic activity of the BQ–Py nanoparticles

**Fig. 7:**
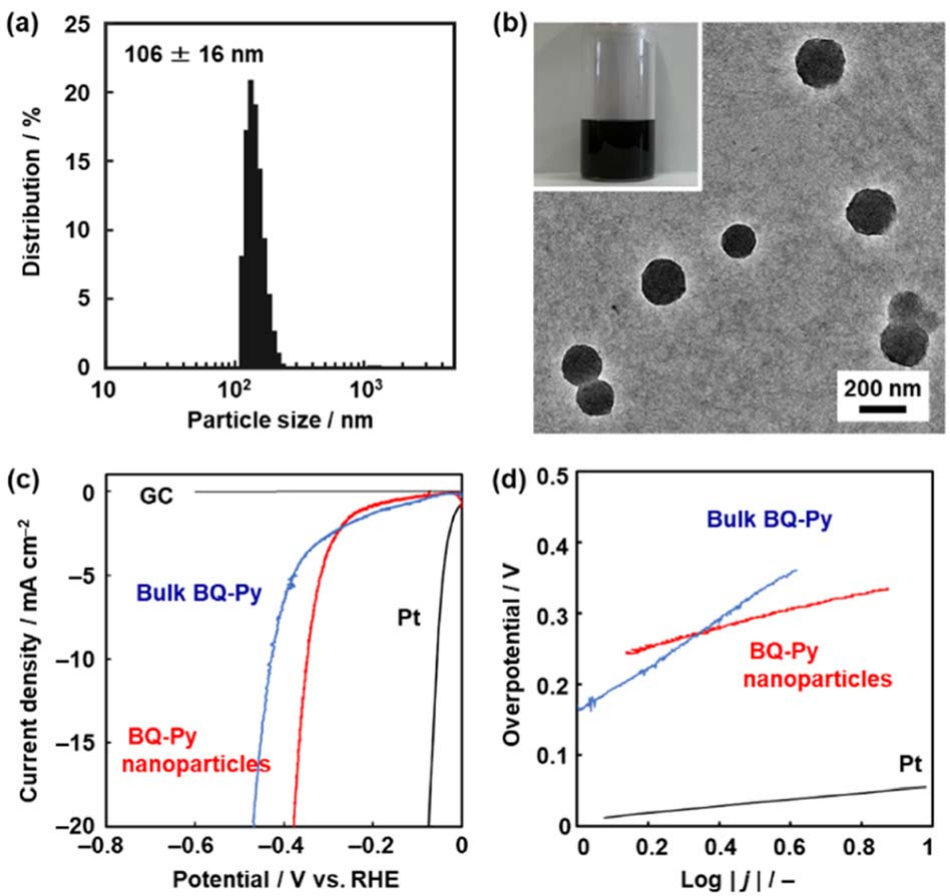
Dispersion of the BQ–Py nanoparticles and their application to HER catalysts. (a) DLS particle-size distribution of the BQ–Py nanoparticles dispersed in acetylacetone. (b) TEM image and photograph of the acetylacetone dispersion liquid (inset). (c and d) LSV curves (c) and Tafel slopes (d) of the BQ–Py bulk particles, BQ–Py nanoparticles, and reference GC and Pt.

The BQ–Py particles 100 nm in size synthesized under standard conditions, *i.e.* pH 2.0, with a monomer concentration of 0.5 mol dm^−3^ were used as an electrocatalyst for the HER because the sample was fully characterized. The BQ–Py nanoparticles were redispersed in organic media under sonication for casting on an electrode (Fig. 7). Prior to the experiments, the dispersibility was studied using the Hansen similarity (solubility) (HSP) parameter distance between the dispersion medium and BQ–Py polymer (Table S4 in the ESI†). In general, the HSP distance is used to estimate the affinity between the soluble polymer and solvent. In our previous work, the parameter was applied as a metric to predict the dispersibility of the nanomaterials in organic media.^52,53^ Here we calculated the HSP distance between the minimum partial molecular structure of BQ–Py and seven organic solvents to prepare a colloidal liquid (Scheme S1 and Table S4 in the ESI†). As the dispersion liquid is used for drop-casting on an electrode, the dispersion media are selected based on both the HSP distance and volatility. The BQ–Py nanoparticles were dispersed in 1,3-dioxolane, acetylacetone, acetone, and dichloromethane. A monodispersed peak with an average diameter of 106 ± 16 nm was observed in acetylacetone using dynamic light scattering (DLS) (Fig. 7a). The particle size was consistent with that measured from the SEM images (Fig. 2b and j). The redispersed BQ–Py nanoparticles around 100 nm in size were observed in the transmission electron microscopy (TEM) image (Fig. 7b). In contrast, larger aggregates formed in the other dispersion media (Fig. S5 in the ESI†).

The BQ–Py nanoparticles were drop-cast on a GC electrode for use as a metal-free electrocatalyst for the HER (Fig. 7c and d). The working electrode was set in a twin beaker cell with a graphite counter and Ag/AgCl reference electrodes. The electrolyte solution was 0.5 mol dm^−3^ H_2_SO_4_. Prior to measurement of the catalytic activity, the BQ–Py nanoparticles on the working electrode were reduced by chronoamperometry (CA) at −0.499 V *vs.* RHE for 5 h to recover the conjugated structure. Then, linear sweep voltammetry (LSV) was carried out in the range of 0 and −0.6 V *vs.* RHE at a scan rate of 5 mV s^−1^ (Fig. 7c). The HER catalytic performance was evaluated by using the overpotential (Δ*E*) at 10 mA cm^−2^ in the LSV curve. The Δ*E* was 336 mV for the BQ–Py nanoparticle (Fig. 7c), whereas the bulk BQ–Py particle several hundred micrometers in size showed Δ*E* = 429 mV (Fig. 7c). The Tafel slope representing the kinetics of the HER was 132 mV dec^−1^ for the nanoparticle and 290 mV dec^−1^ for the bulk particle (Fig. 7d). The catalytic activity was reproducible for five samples (Fig. S6 in the ESI†). The nanoparticles exhibited improved catalytic activity compared with the bulk particles. The high specific surface area of the nanoparticles contributes to improvement of the catalytic activity.

## Conclusions

The morphology and particle size of BQ–Py, an amorphous conjugated polymer network, was controlled by precipitation polymerization in the solution phase. In our previous work, the morphology and size control was not achieved by solid–vapor polymerization. Rapid homogeneous precipitation was achieved by mixing the solutions of BQ and Py. The particle size was controlled in the range of 70 nm and 1 μm by changes in the pH and monomer concentration. The size and morphology control of the BQ–Py amorphous conjugated polymer network was achieved by precipitation polymerization. The resultant BQ–Py nanoparticles exhibited higher activity as a metal-free electrocatalyst for the HER compared with the bulk particles. The results indicate that the size and morphology of other amorphous conjugated polymer networks can be controlled by precipitation polymerization.

## Conflicts of interest

There are no conflicts to declare.

## Supplementary Material

NA-006-D3NA01006F-s001
