# Supplementary material for: Morphology and size control of an amorphous conjugated polymer network containing quinone and pyrrole moieties via precipitation polymerization
Source: Nanoscale Adv. 2023 Dec 27;6(4):1084–90. doi: 10.1039/d3na01006f (PMC10863716; doi:10.1039/d3na01006f)
Supplement: NA-006-D3NA01006F-s001 [file NA-006-D3NA01006F-s001.pdf]

Electronic Supplementary Material (ESI) for Nanoscale Advances.
This journal is © The Royal Society of Chemistry 2023

Electronic Supplementary Information (ESI)

Morphology and size control of an amorphous conjugated polymer network containing quinone and pyrrole moieties via precipitation polymerization

Ryuto Sugiura,[a] Hiroaki Imai,[a] Yuya Oaki*,[a]

[a] Department of Applied Chemistry, Faculty of Science and Technology, Keio University, 3-14-1 Hiyoshi, Kohoku-ku, Yokohama 223-8522, Japan.

*E-mail: oakiyuya@applc.keio.ac.jp

Contents

Experimental methods P. S2
Additional SEM images (Fig. S1) P. S4
Summary of the yield and particle size (Table S1) P. S5
BQ-Py nanoparticles synthesized at the different conditions (Fig. S2) P. S6
^{13}C-MAS-NMR spectrum (Fig. S3) P. S7
UV-Vis-NIR spectrum (Fig. S4) P. S8
Composition of the BQ-Py particles (Tables S2 and S3) P. S9
HSP distance of BQ-Py and organic solvents (Table S4 and Scheme S1) P. S10
Particle-size distributions measured by DLS (Fig. S5) P. S11
Additional LSV curves (Fig. S6) P. S12

Experimental methods

Synthesis of BQ-Py particles in solution phase. BQ monomer (2.0 mmol, TCI 98.0 %) was dissolved (or dispersed for purified water) in 4 cm^3 of solvents, such as purified water, ethanol (Kanto 99.0 %), 2-propanol (TCI 99.5 %), DMF (TCI 99.5 %), THF (Kanto 99.5 %), toluene (Kanto 99.5 %), acetone (Kanto 99.5 %), and ethyl acetate (TCI 98.0 %), in a glass bottle (6 cm^3) at 60 °C. The neat Py liquid (2.0 mmol, TCI 99.0 %) heated at 60 °C was immediately dropped in the stirred solution (or dispersion liquid) containing BQ. The mixture was maintained at 60 °C for 1 h. All these processes were carried out in a drying oven set at 60 °C. The sample bottle was directly dried under vacuum at 200 °C for 16 h to remove the solvent and remaining oligomers.

The pH-adjusted acid solution was prepared using HNO_3 (Kanto 60.0 %), CH_3COOH (Junsei 99.7 %), H_2SO_4 (Kanto 96.0 %), and HCl (Kanto 35.0 %). BQ (2.0 mmol) was dispersed in 4 cm^3 of the pH-adjusted solution at 60 °C. The neat Py liquid (2.0 mmol) was immediately dropped in the stirred dispersion liquid containing BQ. The sample bottle was maintained at 60 °C for 1 h. All these processes were carried out in a drying oven set at 60 °C. The precipitates were collected by centrifugation at 13500 rpm for 15 min. The sample bottles were dried under vacuum at 200 °C for 16 h.

Py (2.0 mmol) was dissolved in 2 cm^3 of the mixed solution of HCl aq. (pH 2.0 or 3.0) and ethanol at 60 °C. BQ (2.0 mmol) dissolved in 2 cm^3 of the mixed solution was separately prepared at 60 °C. The Py-containing solution was mixed with the BQ-containing solution and then maintained at 60 °C for 1 h. All these processes were carried out in a drying oven set at 60 °C The precipitate was collected by centrifugation and then dried under vacuum at the same conditions.

Structural characterization. The morphologies were observed by SEM (JEOL JSM-7100F) operated at 5.0 kV after the ultrathin osmium coating to ensure the conductivity. The structural analyses of the BQ-Py powdered sample were performed by TG (Shimadzu DTG-60) under air atmosphere, FT-IR (Jasco FT/IR 4100) with KBr method, XRD (Bruker D8 Advance) with Cu-Kα radiation, Raman spectroscopy (Renishaw InVia Raman) with the excitation at 532 nm, and ^{13}C solid-state NMR (JEOL JNM-ECZ400R). The reaction behavior in the different solution conditions was analyzed using a spectrophotometer UV-Vis (Jasco V-670). UV-Vis-NIR spectrum of the BQ-Py particle was measured using the same equipment by diffuse-reflectance method.

The dispersion liquid containing BQ-Py powder (15 mg) in 20 cm^3 of organic media, such as acetylacetone (Kanto 99.5 %), 1,3-dioxolane (TCI 98.0 %), dichloromethane (Kanto 99.5 %), and acetone, was prepared using an ultrasonic bath for 15 min and ultrasonic homogenizer for 1 h at room temperature. The particle-size distribution in the dispersion liquid was measured using DLS (Otsuka Electronics ELSZ-2000). The same dispersion liquid was used for the TEM observation (FEI Tecnai G2).

Electrochemical measurement. The above dispersion liquid (15 mm^3) of BQ-Py powder (15 mg) in acetylacetone (20 cm^3) was dropped on a GC electrode without using any binder to prepare the working electrode. The electrode was set in a twin-beaker cell with Ag/AgCl reference and graphite-rod counter electrodes. CA and LSV were measured in 0.5 mol dm^{-3} H_2SO_4 using multichannel electrochemical measurement systems (Hokuto Denko HZ-Pro and Parstat MC).

Additional SEM images

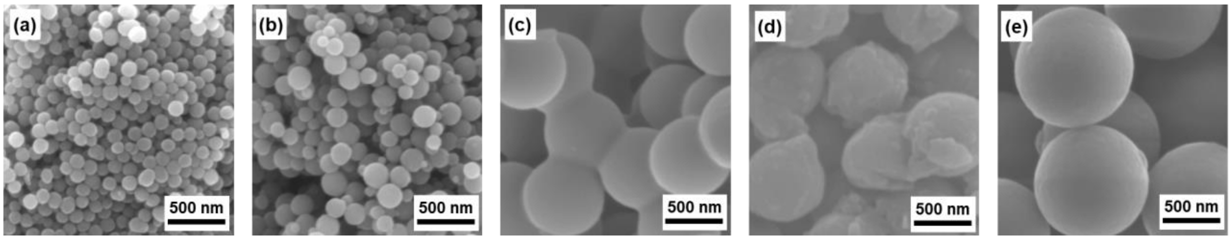

Fig. S1. SEM images of the BQ-Py particles. (a) pH 1.5, 0.5 μmol dm^{-3}. (b) pH 2.5, 0.5 μmol dm^{-3}. (c) pH 3.5, 0.5 μmol dm^{-3}. (d) pH 4.0. 0.25 μmol dm^{-3}. (e) pH 5.0, 0.25 μmol dm^{-3}.

The average particle sizes in Fig. 4j were calculated based on these images and Fig. 4a–i. The data and method were described with Table S1.

Summary of the yield and particle size

Table S1. Yield and particle size of BQ-Py synthesized at the different conditions.

pH	Monomer concentration / µmol dm^{-3}	Yield / %	Particle size / nm
[a] 1.0	0.5	26.1	262 ± 111
[a] 1.5	0.5	32.2	105 ± 22
[a] 2.0	0.5	25.4	112 ± 23
[a] 2.5	0.5	24.9	172 ± 44
[a] 3.0	0.5	31.3	337 ± 61
[a] 3.5	0.5	24.5	672 ± 98
[a] 4.0	0.5	24.6	870 ± 158
[a] 5.0	0.5	22.4	1042 ± 189
[a] 2.0	0.25	18.1	73.5 ± 17
[a] 2.0	1.0	27.7	212 ± 49
[b] 3.0	0.25	30.4	179 ± 55
[b] 3.0	1.0	26.9	573 ± 147
[b] 4.0	0.25	17.5	642 ± 102
[b] 5.0	0.25	19.8	899 ± 265

[a] The sample was prepared in two different batches. The number of the counted particles were 100 in each batch, total number of the samples $N = 200$, for calculation of the average.

[b] The sample was prepared in one batch. The number of the counted particles were 100, total number of the samples $N = 100$, for calculation of the average.

BQ-Py nanoparticles synthesized at the different conditions

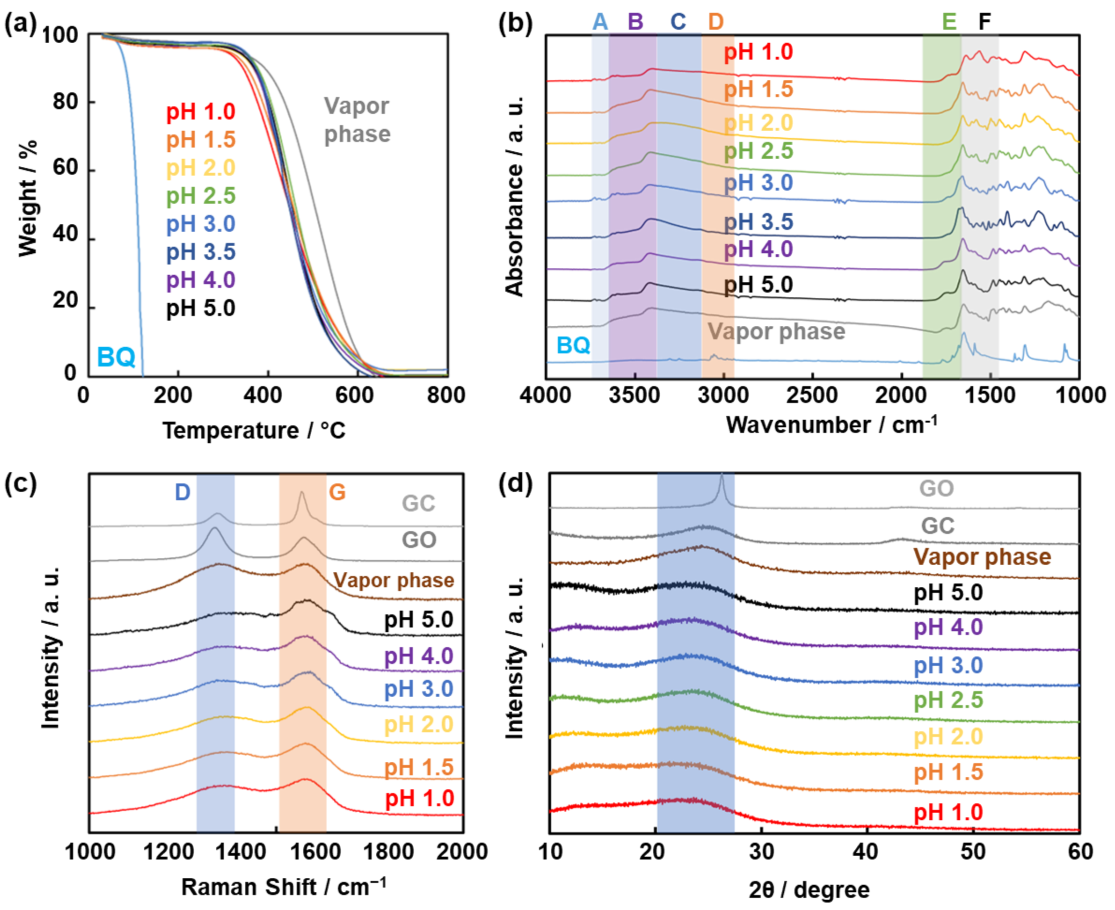

Fig. S2. Structure and composition of the BQ-Py nanoparticles synthesized at pH 1.0–5.0 and their reference samples, such as BQ monomer and BQ-Py prepared by the vapor-phase syntheses in our previous report.[13] (a) TG curves. (b) FT-IR spectra. (c) Raman spectra. (d) XRD patterns.

The BQ-Py network polymers were synthesized in the range of pH 1.0–5.0. Although the similar polymer network structure was obtained in these conditions, the composition was different as shown in Tables S2 and S3 and Figs. 5e and 6. The peak assignments are referred to those in the main text.

^{13}C-MAS-NMR spectrum

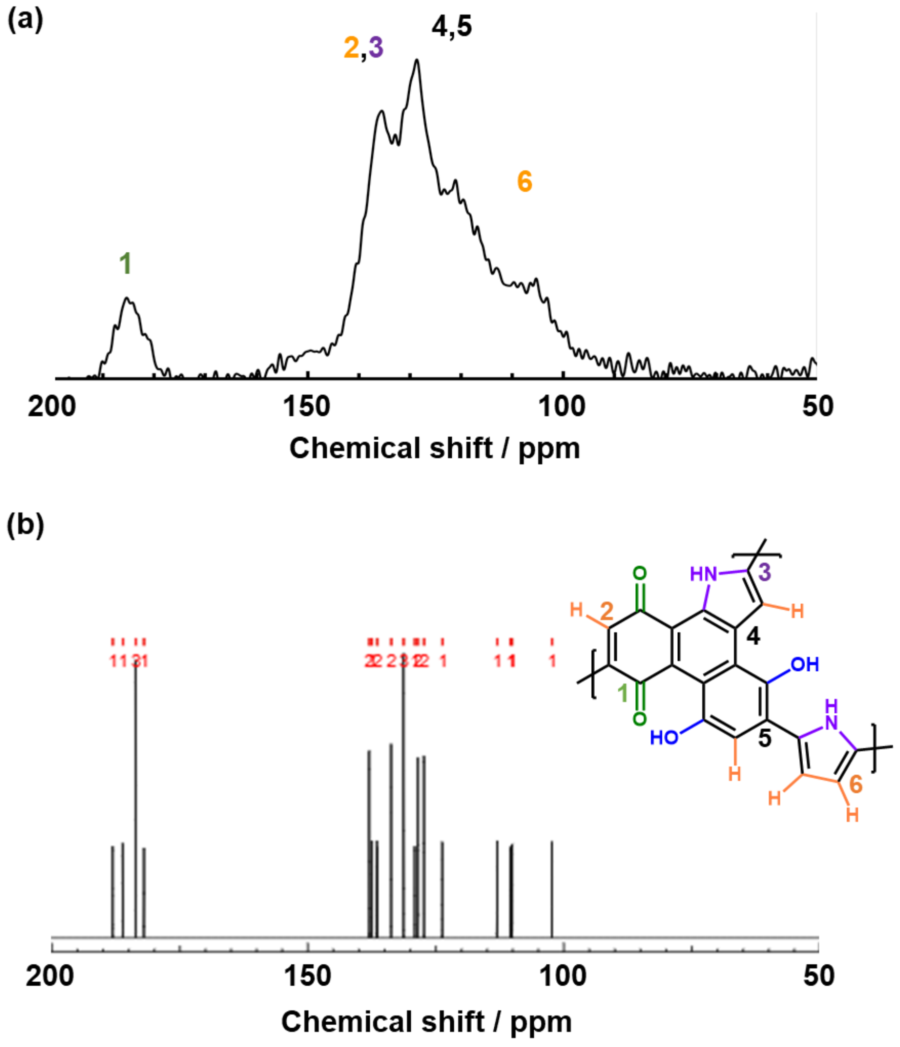

Fig. S3. ^{13}C-MAS-NMR spectra of the BQ-BO nanoparticles synthesized at pH 2.0. (a) Measured spectrum. (b) Calculation simulation of the chemical shift and its unit structure.

The observed spectrum was similar to the cumulated spectrum of the calculation simulation based on the unit structure.

UV-Vis-NIR spectrum

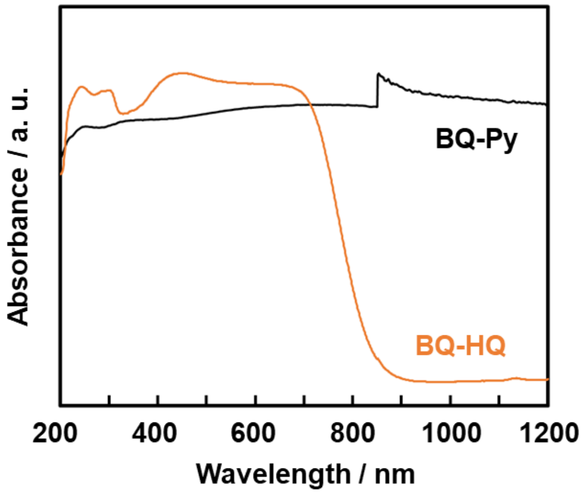

Fig. S4. UV-Vis-NIR spectra of the BQ-Py nanoparticles synthesized at pH 2 (black) and the BQ-HQ charge transfer complex (orange).

The BQ-Py polymer had the absorption in the UV-Vis-NIR range, whereas the absorption edge was observed around 800 nm for the BQ-HQ charge transfer complex. The absorption spectra imply that BQ-Py had the extended conjugated length.

Composition of the BQ-Py particles

Table S2. Measured and calculated compositions of BQ-Py synthesized at pH 1.0–5.0.

	Measured / wt%				Calculated / wt%			
pH	C	H	N	O[a]	C	H	N	O
1.0	67.7	3.00	10.0	19.3	67.9	3.30	9.60	19.2
1.5	67.9	2.79	8.52	20.8	67.7	3.10	8.49	20.7
2.0	66.5	2.81	7.68	23.0	66.9	3.00	7.31	22.8
2.5	68.0	2.66	6.96	22.3	67.9	2.75	6.97	22.4
3.0	67.9	2.78	6.72	22.6	68.0	2.74	6.64	22.6
3.5	67.9	2.95	6.54	22.6	68.2	2.71	6.52	22.6
4.0	67.1	2.60	6.40	23.9	67.3	2.72	6.17	23.8
5.0	67.4	2.75	6.40	23.4	67.5	2.79	6.24	23.4

[a]The weight ratio of O was calculated as that of the other elements except C, H, and N.

Table S3. Estimated composition of the monomer units in the BQ-Py network polymers synthesized at pH 1.0–5.0.

pH / –	*p* / mol%	*q* / mol%	*x* / mol%	*y* / mol%	*w* / mol%	HQ moiety / mol%	*R* [b]
1.0	62.0	23.2	9.39	5.43	27.6	32.3	58.5
1.5	54.6	27.3	11.1	6.91	29.5	30.9	53.0
2.0	45.7	32.0	14.4	7.78	38.1	31.2	47.3
2.5	41.3	33.3	15.7	9.70	27.3	30.1	44.9
3.0	38.6	33.3	17.0	9.35	23.9	20.5	43.7
3.5	36.2	34.7	18.4	9.68	21.8	36.7	42.1
4.0	33.1	36.5	19.8	10.6	34.3	34.3	40.7
5.0	33.3	36.6	20.5	9.49	30.4	39.7	40.7
Nanosheet[a]	24.7	26.3	19.5	14.7	14.7	31.6	40.2

[a] The BQ-Py nanosheet was synthesized in our previous report.[13]

[b] The ratio of the Py moiety to BQ one in the structure was calculated by ($R = (p + x + 2y) / (q + 2x + 3y)$).

The molar ratio of water (w) was calculated from the weight loss lower than 200 °C in the TG curves (Fig. S2). The molar fraction (p, q, x, y: $p + q + x + y = 1$) was estimated from the measured weight ratio of C, H, and N in Table S2. The weight ratio of C, H, and N based on the estimated molar ratio (p, q, x, y) was consistent with the measured value (Table S3).

HSP distance of BQ-Py and organic solvents

Table S4. HSP distance of the BQ-Py unit structure and organic solvents.

Dispersion media	HSP distance / –
Chlorobenzene	12.6
1,3-Dioxolane	10.5
Acetone	13.0
Acetonitrile	12.8
Acetylacetone	10.0
Dichloromethane	12.0
N-methyl-2-pyrrolidone (NMP)	6.9

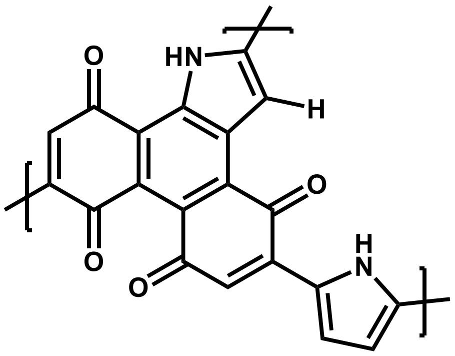

Scheme S1. Structural unit of the BQ-Py polymer for the calculation of the HSP distance.

HSP distance was calculated using a commercial software (HSPiP, version 5.0.03). The smaller value indicates the higher affinity between the two molecules. The unit structure of the BQ-Py polymer as shown in Scheme S1 was assumed to calculate the HSP distance. The dispersion media were selected based on the HSP distance to ensure the dispersion. In addition, the volatility was referred because the dispersion liquid was used for the drop-casting on the electrode. In the present work, acetylacetone, 1,3-dioxolane, dichloroethane, and acetone were selected for the experiments.

Particle-size distributions measured by DLS

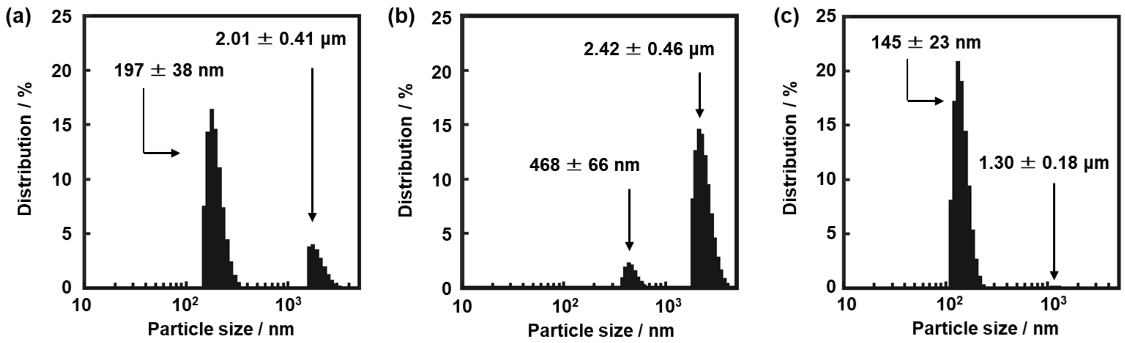

Fig. S5. DLS charts of the BQ-Py nanoparticles dispersed in 1,3-dioxolane (a), dichloromethane (b), and acetone (c).

The smallest particle size was observed in acetylacetone (Fig. 7b). The fact implies that the more homogeneous dispersion is achieved in acetylacetone. Therefore, the electrode was prepared by drop-casting of the dispersion liquid of acetylacetone.

Additional LSV curves

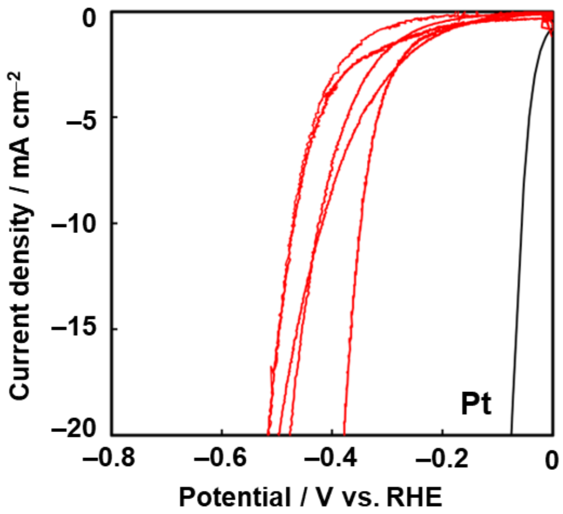

Fig. S6. LSV curves of the BQ-Py nanoparticles on the five different electrodes.

The average overpotential at 10 mA cm^{-2} was 419 ± 41 mV for the five different electrodes.
